# Carbon-ion radiotherapy for clear cell odontogenic carcinomas

**DOI:** 10.1186/s12957-024-03470-x

**Published:** 2024-07-25

**Authors:** Hiroaki Ikawa, Masashi Koto, Kazunori Fugo, Hirotoshi Takiyama, Tetsuro Isozaki, Makoto Shinoto, Shigeru Yamada, Hitoshi Ishikawa

**Affiliations:** QST Hospital, National Institutes for Quantum Science and Technology (QST), 4-9-1 Anagawa, Inage-ku, Chiba-shi, Chiba, 263-8555 Japan

**Keywords:** Charged particle therapy, Carbon-ion radiation therapy, Odontogenic carcinoma

## Abstract

**Background:**

Clear cell odontogenic carcinoma (CCOC) is a rare odontogenic malignant tumor. The standard treatment for CCOC is surgical resection and adjuvant radiotherapy (RT). Radiotherapy is generally considered in inoperable cases. However, there are no reports on definitive RT for CCOC, and the role of RT in patients with inoperable CCOC remains unknown. Therefore, in this report, we present two cases of carbon-ion (C-ion) RT for CCOC.

**Case presentation:**

In case 1, a 73-year-old man with mandibular CCOC presented with recurrence in the inferior temporal fossa after two tumor resections. The tumor was considered inoperable, and C-ion RT (57.6 Gy in 16 fractions) was administered. The tumor remained controlled even after 20 months of C-ion RT; however, the patient died of other causes. In case 2, a 34-year-old man with maxillary CCOC presented with recurrence in the left sinonasal region after two tumor resections. The tumor was considered inoperable, and C-ion RT (64 Gy in 16 fractions) was administered. However, recurrence was observed in the irradiated field 19 months after the treatment. Subsequently, C-ion RT (64 Gy in 16 fractions) was repeated for the recurrent tumors. Seven years and 6 months after the initial irradiation, the tumor remains controlled, and the patient is alive without any unexpected serious adverse events.

**Conclusion:**

C-ion RT may be an effective treatment option for patients with inoperable CCOC.

## Background

Clear cell odontogenic carcinoma (CCOC) is often considered a rare tumor; it was first described by Hansen in 1985 [1]. Formerly known as a clear cell odontogenic tumor, this locally aggressive benign tumor was renamed as a CCOC in the World Health Organization classification of 1992 [2] and categorized as a malignant tumor after this classification was revised in 2005 [3]. However, it is still considered a malignant tumor according to the 2024 classification [4]. CCOC is a high-grade odontogenic malignant tumor with approximately 117 cases reported so far [4, 5]. However, its incidence remains unclear. The mandible is the most common site of origin; CCOC occurs in the mandible three times as frequently as the maxilla, with 43% of all lesions arising in the posterior body and lower ramus [6]. CCOCs vary in behavior, from indolent tumors to frequently recurring tumors. Recurrence has been reported in 52.4% of cases [5]. The tumors have metastasized in approximately 12% of reported cases, usually to the cervical lymph nodes and lungs and less frequently to the bone [4]. Metastases are rare at the time of presentation, and the outcome in 15% of the cases is death, with a median survival of 14 years. Furthermore, recurrence and metastasis can develop after several years [6, 7].

There are no treatment guidelines for CCOC due to its rarity. The standard treatment in reported cases is complete surgical resection [5, 7]. Adjuvant radiotherapy has no defined role, but it may be appropriate for patients with soft tissue extension, aggressive growth, or incomplete surgical margins [7]. Radiotherapy (RT) is usually considered for inoperable cases [8, 9]; however, no definitive RT has been reported for CCOC cases [7]. Furthermore, the radiosensitivity of CCOCs and the role of radiotherapy in unresectable CCOCs remain unknown. Carbon-ions (C-ions) provide higher linear energy transfer and larger relative biological effectiveness (RBE) than photons [10]. Additionally, C-ion RT provides better dose distribution than conventional photon therapy because of its particle nature; the weight of the particles reduces their lateral scattering [10], reducing the dose to risk organs and safely concentrating the higher doses on the target organ, which is not possible with conventional RT. This offers a greater possibility of tumor control, even in radioresistant non-squamous cell carcinomas (SCCs). In clinical studies, C-ion RT showed therapeutic efficacy in patients with non-SCC, such as salivary gland carcinoma and mucosal melanoma [11, 12]. Therefore, C-ion RT may have therapeutic potential for CCOC as a non-SCC; however, this has not yet been reported. Thus, in this case report, we report two cases of CCOCs treated with C-ion RT at our institution.

### C-ion RT

Detailed target delineation, treatment planning, computed tomography (CT) examinations, and immobilization devices for C-ion RT have been previously described [12, 13]. The clinical and planning target volumes for the initial irradiation and re-irradiation cases were determined based on the reports of Ikawa et al. [12] and Hayasi et al. [13], respectively. The C-ion doses were expressed as RBE-weighted doses based on the modified microdosimetric kinetic model [14] and defined as the physical dose multiplied by the C-ion RBE [15].

### Dose-volume histogram analysis for the organ at risk

Dose-volume histogram (DVH) parameters were calculated using MIM Maestro version 6.8.7. (MIM Software Inc., Cleveland, OH, USA).

### Evaluation and follow-up examination

Follow-up examinations included CT or magnetic resonance imaging (MRI) and endoscopic examinations every 2–3 months for the first 2 years and every 3–6 months after that. Acute and late reactions in normal tissues were classified following the National Cancer Institute’s Common Terminology of Criteria for Adverse Effects (version 4.0) [16]. Tumor response was evaluated following the Response Evaluation Criteria in Solid Tumors guidelines (version 1.1).

## Case presentation

### Case 1

**Fig. 1:**
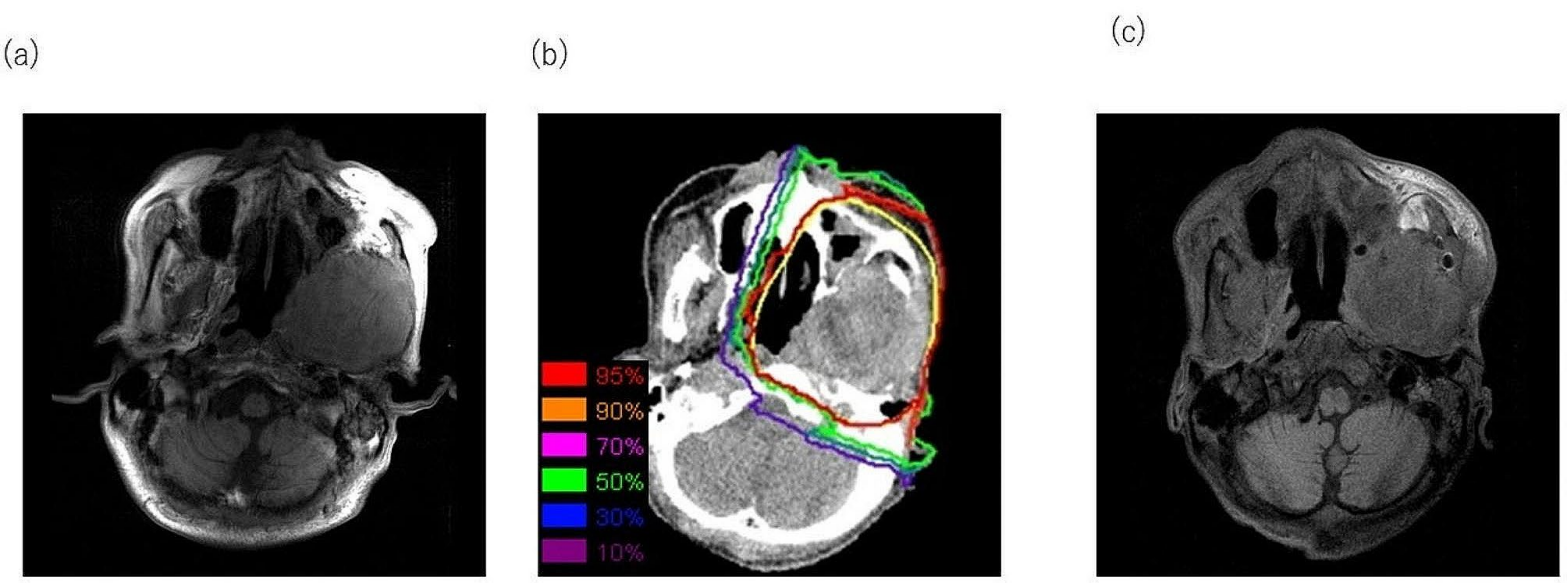
Carbon-ion radiotherapy for mandibular clear cell odontogenic carcinoma (**a**) Extensive clear cell odontogenic carcinoma originating from the left mandible: axial, T2 weighted magnetic resonance imaging (MRI) (**b**) Dose distribution of carbon-ion radiotherapy, with a prescribed dose of 57.6 Gy in 16 fractions The isodose lines correspond to 95%, 90%, 70%, 50%, 30%, and 10% dose areas. Representative computed tomography images are used to delineate planning target volume (yellow)(**c**) Twenty months after carbon-ion radiotherapy: no tumor growth or recurrence and stable disease in axial, contrast-enhanced T1 weighted MRI

A 73-year-old man with an extensive CCOC relapse was referred to our institution for C-ion RT. The patient had undergone surgical therapy for the right mandible 17 years before presenting at our hospital. However, no diagnosis was made at that time. Thirteen years postoperatively, the patient noticed painless swelling of the alveolar mucosa of the left anterior mandible, which rapidly enlarged and was ulcerated. A biopsy was performed, and the patient was initially diagnosed with mucoepidermoid carcinoma. Clinically, the mandibular tumor recurred, and no metastases to regional lymph nodes or other organs were observed. Furthermore, segmental resection of the mandible and reconstruction of the rectus abdominis with myocutaneous and deltopectoral flaps were performed. Finally, the mandibular tumor was confirmed to be a CCOC. Muramatsu et al. have reported the pathological findings and clinical history of this case [17]. Three years and 8 months after the second surgery, local recurrence was observed on a follow-up MRI scan. T1-weighted MRI showed a 67 mm (L), 69 mm (W), and 60 mm (H) low-intensity recurrent tumor located at the intratemporal fossa extending to the cranial base (Fig. 1a). Complete resection was difficult, and C-ion RT was recommended based on interdisciplinary discussions. C-ion RT using the passive irradiation method was administered at 57.6 Gy in 16 fractions (Fig. 1b) and was completed as scheduled. During irradiation, grade 3 mucositis and grade 2 dermatitis appeared, but they improved with conservative treatment. Twenty months after irradiation, no recurrence was observed (Fig. 1c). Furthermore, no lymph nodes or distant metastases were observed; however, the patient died from other diseases. Regarding late adverse events, grade 1 dermatitis persisted, but no osteoradionecrosis was observed. C-ion RT partially irradiated high doses of the reconstructed tissue; however, no flap loss or wound dehiscence was observed.

### Case 2

**Fig. 2:**
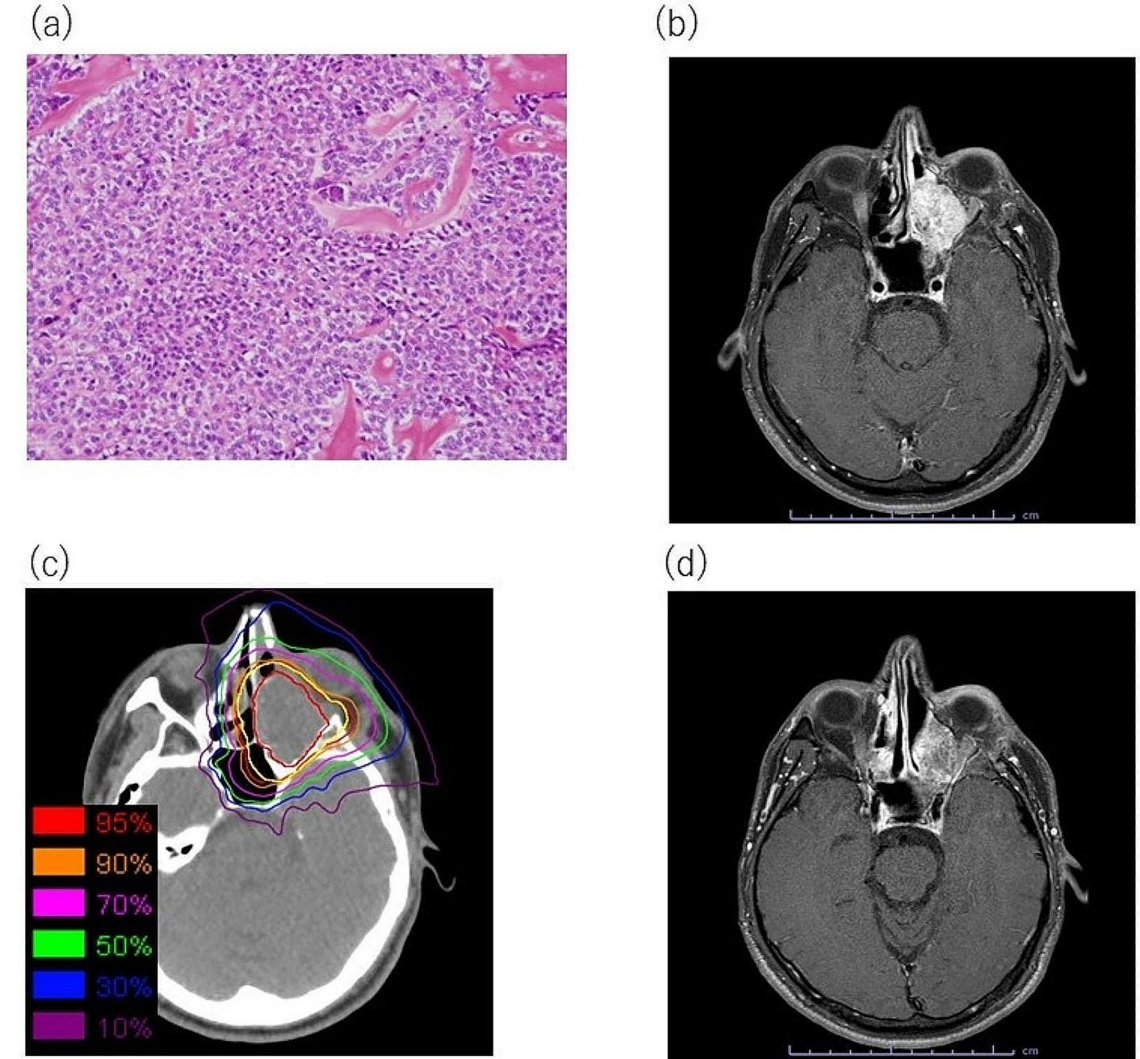
Initial carbon-ion radiotherapy for maxillary clear cell odontogenic carcinoma (**a**) Histopathological findings using hematoxylineosin staining: this specimen is of the initial surgical resection in 2013. The tumor shows predominantly clear to faintly eosinophilic cytoplasm, well-demarcated cell membranes, and irregular, small, dark-staining nuclei (**b**) Recurrence of clear cell odontogenic carcinoma originating from the left maxilla: axial, contrast-enhanced T1 weighted magnetic resonance imaging (MRI) (**c**) Dose distribution of initial carbon-ion radiotherapy, with a prescribed dose of 64 Gy in 16 fractions. The isodose lines correspond to 95%, 90%, 70%, 50%, 30%, and 10% dose areas. Representative computed tomography images are used to delineate gross tumor volume (red) and plan target volume (yellow) (**d**) Fifteen months after carbon-ion radiotherapy, no tumor growth or recurrence is observed, and the patient has a stable disease: axial, contrast-enhanced T1 weighted MRI.

**Fig. 3:**
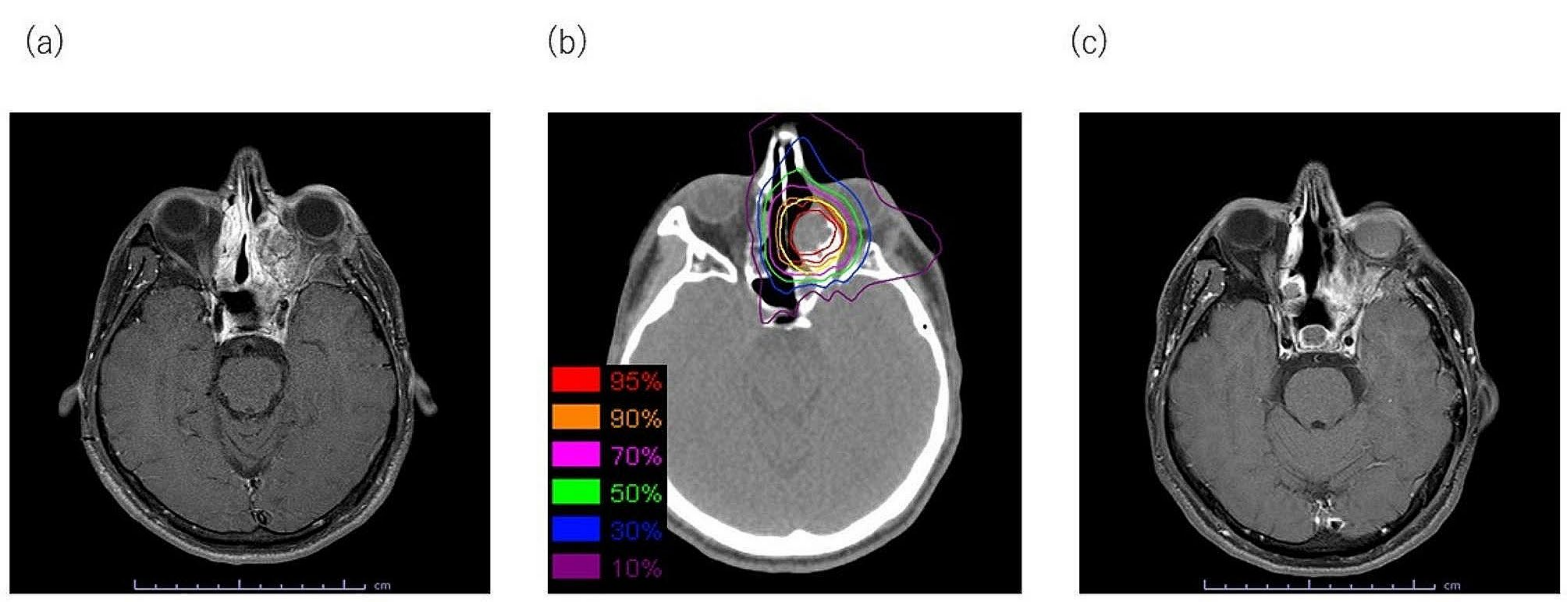
Re-irradiation using carbon-ion radiotherapy for recurrent maxillary clear cell odontogenic carcinoma (**a**) Nineteen months after carbon-ion radiotherapy: The tumor is enlarged and diagnosed as recurrent in axial, contrast-enhanced T1 weighted magnetic resonance imaging (MRI).(**b**) Dose distribution of re-carbon-ion radiotherapy, with a prescribed dose of 64 Gy in 16 fractions: The isodose lines correspond to 95%, 90%, 70%, 50%, 30%, and 10% dose areas. Representative computed tomography images are used for the delineation of gross tumor volume (red) and planning target volume (yellow) (**c**) Six years after re-carbon-ion radiotherapy: The tumor do not show growth or recurrence, and the patient has a stable disease in axial, contrast-enhanced T1 weighted MRI.

A 34-year-old man with a CCOC relapse was referred to our hospital for C-ion RT. The patient presented to the dental clinic with left maxillary tooth pain 3 years and 6 months before presenting to our hospital. The cause of the pain was unknown, and the patient was referred to the Oral Surgery Division of the hospital. A CT scan revealed a neoplastic lesion in the left maxilla. A biopsy was performed, and odontogenic carcinoma was suspected. Partial maxillectomy with tumor resection and reconstruction using a rectus abdominis musculocutaneous flap were performed, and the pathological diagnosis was CCOC (Fig. 2a). One year and 10 months after the initial surgery, a local recurrence was observed, and tumor resection was performed. However, 1 year and 6 months after the second surgery, contrast-enhanced T1-weighted MRI showed a 34 mm (L), 32 mm (W), and 34 mm (H) recurrence extending to the left orbital apex (Fig. 2b). Complete resection was difficult. C-ion RT was recommended based on interdisciplinary discussions. C-ion RT using the scanning irradiation method was administered at 64 Gy in 16 fractions (Fig. 2c) and was completed as scheduled. During irradiation, grade 2 mucositis and grade 1 dermatitis developed, and they improved with conservative treatment. In this C-ion RT plan, DVH analysis for the organ at risk was as follows: dose received at 20% volume (D20%) = 64.60 Gy for the optic nerve, volume receiving 40 Gy (V40) = 3.24 cm^3^, and maximum dose (Dmax) = 59.68 Gy for the eyeball. Subsequently, the tumors tended to shrink (Fig. 2d). One year and 7 months after C-ion RT, a recurrent lesion was found in the irradiated field (Fig. 3a). A treatment strategy was discussed at the Cancer Board. The patient was treated with a C-ion RT re-irradiation (re-C-ion RT) using the scanning irradiation method, administered at 64 Gy in 16 fractions (Fig. 3b). During irradiation, grade 2 mucositis and grade 1 dermatitis developed and were managed using conservative treatment. The DVH analysis for the organ at risk during this re-C-ion RT plan was as follows: D20% = 42.74 Gy for the optic nerve, V40 = 0.36 cm^3^, and Dmax = 54.09 Gy for the eyeball. Seven years and 6 months after the initial irradiation, no local recurrence or distant metastasis has been observed, and the patient is still alive (Fig. 3c). Regarding late adverse events, a grade 4 optic nerve disorder on the affected side was observed 2 years and 10 months after the initial irradiation. However, the right visual function on the healthy side remained normal. Intraocular/vitreous hemorrhage was also observed 3 years and 9 months after the initial irradiation, but the eyeball could be preserved without pain symptoms (Fig. 3c). There was no evidence of osteoradionecrosis. Shrinkage of the grafted tissue was observed in the reconstructed tissue; however, no flap loss or wound dehiscence was observed.

## Discussion

To date, no effective treatment has been reported for cases of inoperable CCOC. Therefore, it is clinically essential to provide a definitive treatment to ensure therapeutic efficacy. To our knowledge, this is the first report on CCOC treatment with RT. We showed that C-ion RT could offer acceptable adverse events and therapeutic efficacy for inoperable CCOCs, although there have been cases of re-C-ion RT. Therefore, C-ion RT should be considered as the definitive treatment for inoperable CCOCs.

Local control was obtained in case 1, with a prescribed dose of 57.6 Gy. However, in case 2, local recurrence occurred after C-ion RT with a prescribed dose of 64 Gy. Therefore, the patient was re-irradiated with C-ion RT at 64 Gy and has been under local control for 7 years and 6 months. We administered two prescribed doses of 57.6 Gy and 64 Gy in 16 fractions to patients 1 and 2 respectively, for the same pathology, based on the findings of a phase I/II trial conducted between 1994 and 1997 [18], in which a dose of 64 Gy was generally prescribed and a dose of 57.6 Gy was recommended when wide areas of skin or mucosa were present in the target volume. In case 1, 57.6 Gy was selected because the target was widely spread in the skin and oral mucosa. Conversely, as there was no skin or oral mucosa in close proximity, a dose of 64 Gy was administered to patient 2. Currently, doses of 64.0 Gy are more commonly employed because advances in treatment techniques such as scanning irradiation methods [19] and rotating gantries [20] have enabled skin and mucosal sparing. As a result, local control was achieved in both cases; however, the optimal dose and fractionation of CCOCs are controversial. Therefore, it is necessary to increase the number of cases and consider the prescribed doses in the future. Additionally, it may be possible to safely treat CCOC using conventional photon RT with advances in treatment techniques such as intensity-modulated RT, although the efficacy is unknown owing to no available reports of photon RT alone for CCOC. Further studies are expected in the future.

Three reports have been published on charged-particle therapy for odontogenic carcinomas [21–23]. Treatment with C-ion RT has been reported for ameloblastic carcinomas (AC), which have the same odontogenic malignancy as CCOC [21]. Jensen et al. reported that C-ion RT with 60 Gy in 20 fractions was effective for inoperable AC cases, although the follow-up period was only 3 months [21]. There are also reports that proton beam therapy (PBT) is effective for AC [22, 23]. Yamagata et al. reported hypofractionated PBT at a dose of 69 Gy in 23 fractions in patients with AC who survived for more than 5 years without any evidence of recurrence or side effects [22]. Takayama et al. reported that PBT (71.4 Gy in 32 fractions) combined with a continuous intra-arterial infusion of cisplatin (40 mg/m^2^) and docetaxel (8 mg/m^2^) for AC resulted in 94 months of survival without relapse or metastasis [23]. The radiosensitivities of AC and CCOC are unknown; however, they are odontogenic carcinomas that may be similar. Therefore, owing to the effectiveness of C-ion RT and PBT in treating AC, therapeutic efficacy using C-ion RT and PBT can be achieved for CCOC cases.

The patient in case 2 received re-C-ion RT for recurrence after C-ion RT and has been in local control for 7 years and 6 months. Hayashi et al. [13] reported on using re-C-ion RT for recurrent head and neck malignancies after C-ion RT. The 2-year local control, locoregional control, and progression-free and overall survival rates were 40.5, 33.5%, 29.4%, and 59.6%, respectively. Re-C-ion RT may be expected to achieve efficacy against CCOC recurrence after C-ion RT. Hayashi et al. also reported that serious late adverse events of grade 3 or higher occurred in 37.5% of patients, including grade 5 central nervous system necrosis in 2% of patients who underwent re-C-ion RT [13]. Therefore, indications for re-irradiation should be carefully considered.

In case 2, a grade 4 optic nerve disorder and intraocular/vitreous hemorrhage were observed. Hasegawa et al. reported that a dose of < 60 Gy at D20% of the optic nerve volume was an independent risk factor for optic nerve disorders after C-ion RT [24]. Furthermore, Nachankar et al. reported that V40 ≥ 0.83 cm3 and Dmax ≥ 54.75 Gy for the eyeball were considered risk factors after C-ion RT for intraocular/vitreous hemorrhage [25]. In this case, the dose to the organs at risk in the initial irradiation and re-irradiation was D20% = 64.60 Gy and 42.74 Gy for the optic nerve, V40 = 3.24 cm3 and 0.36 cm3, and Dmax = 59.68 Gy and 54.09 Gy for the eyeball, respectively. The irradiated doses were higher than the reported values, which may have inevitably led to the development of adverse events. However, owing to the excellent dose distribution of C-ion RT [10], no visual dysfunction occurred on the healthy side.

The clinically significant late adverse event for head and neck cancers after C-ion RT is osteoradionecrosis. None of our two patients had this event. Sasahara et al. reported that a maxillary volume receiving > 50 Gy and the presence of teeth within the planning target volume are risk factors for maxillary osteoradionecrosis [26]. In addition, for mandibular osteoradionecrosis, doses of 30 Gy to the mandible and teeth are the most significant risk factors [27]. The primary locations in the two CCOC cases, one in the mandible and the other in the maxilla, were both recurrent lesions after tumor resection with osteotomy. The jawbone on the primary side had already been resected in both cases before C-ion RT; therefore, there were no jawbones or teeth associated with the risk of osteoradionecrosis. Thus, the patient was considered to have had a good clinical outcome without osteoradionecrosis.

## Conclusion

Report of these cases demonstrates the advantages of C-ion RT for unresectable CCOCs, with good treatment results and acceptable side effects. It also showed that local control with C-ion RT may lead to long-term survival. Therefore, C-ion RT may be an effective treatment option for inoperable CCOCs, although large-sample studies are needed to clarify its efficacy.

## Data Availability

No datasets were generated or analysed during the current study.

## Abbreviations

AC: Ameloblastic carcinomas
CCOC: Clear cell odontogenic carcinoma
C-ion: Carbon-ion
D20%: Dose received at 20% volume
Dmax: Maximum dose
DVH: Dose-volume histogram
PBT: Proton beam therapy
RBE: Relative biological effectiveness
Re-C-ion RT: Carbon-ion radiotherapy re-irradiation
RT: Radiotherapy
SCC: Squamous cell carcinoma
V40: Volume receiving 40 Gy

## References

[1] Hansen LS, Eversole LR, Green TL, Powell NB. Clear cell odontogenic tumor—a new histologic variant with aggressive potential. Head Neck Surg. 1985;8:115–23. 10.1002/hed.2890080208, PMID: 4077550. 10.1002/hed.2890080208 4077550

[2] Le Charpentier Y. Classification des tumeurs odontogènes;14. 2ème édition. OMS [Classification of odontogenic tumors, 2nd edition (WHO, 1992)]. Ann Pathol., pp. 55–7. French. PMID: 8155198; 1994. 8155198

[3] Barnes L, Eveson JW, Reichart P, editors. World Health Organization Classification of Tumours. Pathology and genetics of head and neck tumours. Lyon: IARC Press. World Health Organization; 2005. p. 292.

[4] WHO Classification of Tumours Editorial Board. World Health Organization classification of Tumorus, head and neck tumours part A. 4th ed. Lyon: IARC; 2024. pp. 369–70.

[5] Labrador AJP, Marin NRG, Valdez LHM, Valentina MP, Sanchez KBT, Ibazetta KAR, et al. Clear cell odontogenic carcinoma a systematic review. Head Neck Pathol. 2022;16:838–48. 10.1007/s12105-021-01383-9. [Epub ahead of print]. PMID: 34618301. 34618301 10.1007/s12105-021-01383-9 PMC9424403

[6] Loyola AM, Cardoso SV, de Faria PR, Servato JPS, Barbosa de Paulo LF, Eisenberg ALA et al. Clear cell odontogenic carcinoma: report of 7 new cases and systematic review of the current knowledge. Oral Surg Oral Med Oral Pathol Oral Radiol. 2015;120:483–96. 10.1016/j.oooo.2015.06.005. Epub Jun 15 2015. PMID: 26232924. 10.1016/j.oooo.2015.06.005 26232924

[7] Ebert CS Jr, Dubin MG, Hart CF, Chalian AA, Shockley WW. Clear cell odontogenic carcinoma: a comprehensive analysis of treatment strategies. Head Neck. 2005;27:536–42. 10.1002/hed.20181, PMID: 15772956. 10.1002/hed.20181 15772956

[8] National Comprehensive Cancer Network practice guidelines in. oncology. version 1.2023, p, p. ADV-1–ADV-3. https://www.nccn.org/professionals/physician_gls/f_guidelines.asp. Accessed Apr 28, 2023.

[9] Ikawa H, Sato H, Takayama K, Takeda D, Suzuki T, Yuasa H, et al. Is chemoradiotherapy more effective than radiotherapy alone in patients with primary unresectable locally advanced oral cancer without distant metastases? Systematic review and meta-analysis based on the GRADE approach. J Oral Maxillofac Surg Med Pathol. 2024;36:259–65. 10.1016/j.ajoms.2023.08.010. 10.1016/j.ajoms.2023.08.010

[10] Kamada T. The characteristics of carbon-ion radiotherapy. In: Tsujii H, Kamada T, Shirai T, Noda K, Tsuji H, Karasawa K, editors. Carbon-ion radiotherapy: principles, practices, and treatment planning. Tokyo: Springer Science and Business Media; 2013.

[11] Ikawa H, Koto M, Demizu Y, Saitoh JI, Suefuji H, Okimoto T, et al. Multicenter study of carbon-ion radiation therapy radiotherapy for nonsquamous cell carcinomas of the oral cavity. Cancer Med. 2019;8:5482–91. 10.1002/cam4.2408. Epub Aug 1 2019. PMID. PMCID: PMC6745861. 31369213 10.1002/cam4.2408 PMC6745861

[12] Ikawa H, Koto M, Hayashi K, Tonogi M, Takagi R, Nomura T, et al. Feasibility of carbon-ion radiotherapy for oral non-squamous cell carcinomas. Head Neck. 2019;41:1795–803. 10.1002/hed.25618. 30676669 10.1002/hed.25618 PMC6590439

[13] Hayashi K, Koto M, Ikawa H, Hagiwara Y, Tsuji H, Ogawa K, et al. Feasibility of re-irradiation using carbon ions for recurrent head and neck malignancies after carbon-ion radiotherapy. Radiother Oncol. 2019;136:148–53. 10.1016/j.radonc.2019.04.007. Epub Apr 19 2019. PMID: 31015117. 31015117 10.1016/j.radonc.2019.04.007

[14] International Commission on Radiation Units and Measurements. Report 93: prescribing, recording, and reporting light ion beam therapy. J ICRU. 2016;16:1–211.

[15] Inaniwa T, Kanematsu N, Matsufuji N, Kanai T, Shirai T, Noda K, et al. Reformulation of a clinical-dose system for carbon-ion radiotherapy treatment planning at the National Institute of Radiological Sciences, Japan. Phys Med Biol. 2015;60:3271–86. 10.1088/0031-9155/60/8/3271. 25826534 10.1088/0031-9155/60/8/3271

[16] Common terminology criteria for adverse events (CTCAE). version 4.0; published 2009. rev. version 4.03 Accessed Jun 14, 2010. https://evs.nci.nih.gov/ftp1/CTCAE/CTCAE_4.03/CTCAE_4.03_2010-06-14_QuickReference_8.5x11.pdf. Accessed Jul 12, 2019.

[17] Muramatsu T, Hashimoto S, Inoue T, Shimono M, Noma H, Shigematsu T. Clear cell odontogenic carcinoma in the mandible: histochemical and immunohistochemical observations with a review of the literature. J Oral Pathol Med. 1996;25:516–21. 10.1111/j.1600-0714.1996.tb00308.x, PMID: 8959562. 10.1111/j.1600-0714.1996.tb00308.x 8959562

[18] Mizoe JE, Tsujii H, Kamada T, Matsuoka Y, Tsuji H, Osaka Y et al. Dose escalation study of carbon ion radiotherapy for locally advanced head-and-neck cancer. Int J Radiat Oncol Biol Phys. 2004;60:358–64. 10.1016/j.ijrobp.2004.02.067, PMID: 15380567. 10.1016/j.ijrobp.2004.02.067 15380567

[19] Inaniwa T, Furukawa T, Kanematsu N, Mori S, Mizushima K, Sato S et al. Evaluation of hybrid depth scanning for carbon-ion radiotherapy. Med Phys. 2012;39:2820–5. 10.1118/1.4705357, PMID: 22559653. 10.1118/1.4705357 22559653

[20] Bhattacharyya T, Koto M, Ikawa H, Hayashi K, Hagiwara Y, Makishima H, et al. First prospective feasibility study of carbon-ion radiotherapy using compact superconducting rotating gantry. Br J Radiol. 2019;92:20190370. 10.1259/bjr.20190370. Epub Jul 24 2019. PMID: 31317764, PMCID: PMC6849685. 31317764 10.1259/bjr.20190370 PMC6849685

[21] Jensen AD, Ecker S, Ellerbrock M, Nikoghosyan A, Debus J, Münter MW. Carbon ion therapy for ameloblastic carcinoma. Radiat Oncol. 2011;6:13. 10.1186/1748-717X-6. 13, PMID. PMCID: PMC3038940. 21294917 10.1186/1748-717X-6 PMC3038940

[22] Yamagata K, Ishikawa H, Saito T, Bukawa H. Proton Beam therapy for ameloblastic carcinoma of the maxilla: report of a rare case. J Oral Maxillofac Surg. 2019;77:e2271–5. 10.1016/j.joms.2018.08.014. Epub Aug 24 2018. PMID: 30240599. 10.1016/j.joms.2018.08.014 30240599

[23] Takayama K, Nakamura T, Takada A, Kato T, Sakuma H, Mitsudo K, et al. Proton Beam therapy combined with retrograde intra-arterial infusion chemotherapy for an extremely rapid growing recurrent ameloblastic carcinoma: a case report. Mol Clin Oncol. 2020;13:34. 10.3892/mco.2020.2104. Epub Jul 30 2020. PMID. PMCID: PMC7412713. 32802330 10.3892/mco.2020.2104 PMC7412713

[24] Hasegawa A, Mizoe JE, Mizota A, Tsujii H. Outcomes of visual acuity in carbon ion radiotherapy: analysis of dose-volume histograms and prognostic factors. Int J Radiat Oncol Biol Phys. 2006;64:396–401. 10.1016/j.ijrobp.2005.07.298. Epub Sep 22 2005. PMID: 16182466. 10.1016/j.ijrobp.2005.07.298 16182466

[25] Nachankar A, Musha A, Kubo N, Kawamura H, Okano N, Sato H, et al. Dosimetric analysis of intraocular hemorrhage in nonsquamous head and neck cancers treated with carbon-ion radiotherapy. Radiother Oncol. 2022;170:143–50. 10.1016/j.radonc.2022.02.032. Epub Mar 4 2022. PMID: 35257851. 35257851 10.1016/j.radonc.2022.02.032

[26] Sasahara G, Koto M, Ikawa H, Hasegawa A, Takagi R, Okamoto Y, et al. Effects of the dose-volume relationship on and risk factors for maxillary osteoradionecrosis after carbon ion radiotherapy. Radiat Oncol. 2014;9:92. 10.1186/1748-717X. -9-92, PMID. PMCID: PMC3992144. 24708583 10.1186/1748-717X PMC3992144

[27] Musha A, Shimada H, Kubo N, Kawamura H, Okano N, Sato H et al. Clinical features and dosimetric evaluation of carbon ion radiation-induced osteoradionecrosis of mandible in head and neck tumors. Radiother Oncol. 2021;161:205–10. 10.1016/j.radonc.2021.06.022. Epub Jun 18 2021. PMID: 34147522. 10.1016/j.radonc.2021.06.022 34147522

